# Clotrimazole inhibits growth of multiple myeloma cells in vitro via G0/G1 arrest and mitochondrial apoptosis

**DOI:** 10.1038/s41598-024-66367-5

**Published:** 2024-07-04

**Authors:** Yang Song, Hui Zhang, Jie Geng, Haoran Chen, Yang Bo, Xuechun Lu

**Affiliations:** 1 Chinese People’s Liberation Army Medical School, Beijing, 100853 China; 2 School of Basic Medicine, Shanxi Medical University, Taiyuan, 030000 China; 3 Department of Hematology, Second Medical Center, People’s Liberation Army General Hospital, Beijing, 100853 China

**Keywords:** Clotrimazole, Multiple myeloma, Cell cycle, Reactive oxygen species, Apoptosis, Cancer, Medical research

## Abstract

Patients with multiple myeloma (MM) experience relapse and drug resistance; therefore, novel treatments are essential. Clotrimazole (CTZ) is a wide-spectrum antifungal drug with antitumor activity. However, CTZ’s effects on MM are unclear. We investigated CTZ’s effect on MM cell proliferation and apoptosis induction mechanisms. CTZ’s effects on MM.1S, NCI- H929, KMS-11, and U266 cell growth were investigated using Cell Counting Kit-8 (CCK-8) assay. The apoptotic cell percentage was quantified with annexin V-fluorescein isothiocyanate/7-amino actinomycin D staining. Mitochondrial membrane potential (MMP) and cell cycle progression were evaluated. Reactive oxygen species (ROS) levels were measured via fluorescence microscopy. Expression of apoptosis-related and nuclear factor (NF)-κB signaling proteins was analyzed using western blotting. The CCK-8 assay indicated that CTZ inhibited cell proliferation based on both dose and exposure time. Flow cytometry revealed that CTZ decreased apoptosis and MMP and induced G0/G1 arrest. Immunofluorescence demonstrated that CTZ dose-dependently elevated in both total and mitochondrial ROS production. Western blotting showed that CTZ enhanced Bax and cleaved poly ADP-ribose polymerase and caspase-3 while decreasing Bcl-2, p-p65, and p-IκBα. Therefore, CTZ inhibits MM cell proliferation by promoting ROS-mediated mitochondrial apoptosis, inducing G0/G1 arrest, inhibiting the NF-κB pathway, and has the potential for treating MM.

## Introduction

Multiple myeloma (MM) is a serious hematologic malignancy that causes an abnormal proliferation of plasma cells in the bone marrow and an excessive production of monoclonal immunoglobulins or light chains^1,2^. MM is responsible for approximately 17% of all hematologic malignancies^3^. Clinical manifestations include extensive bone destruction, anemia, recurrent infections, hypercalcemia, and renal failure^4^. According to the Global Cancer Observatory database, approximately 180,000 new cases of MM were diagnosed in 2020, and 117,000 deaths occurred worldwide, posing a significant health and socioeconomic burden^5^ Over the past 10–15 years, advances in treatment, such as new drugs, have dramatically enhanced the survival rate of individuals with MM^6^. However, no cure is currently available. Patients experience relapse and drug resistance^7^. Therefore, identifying novel drug treatments for MM is essential.

Clotrimazole (CTZ) is a synthetic imidazole antifungal medication with a wide spectrum of activity that is used in the management of *Candida albicans* and a variety of other fungal infections^8^. This drug targets ergosterol biosynthesis, disrupting fungal cell wall structure and function by causing cell wall leakage^9^. CTZ also has antibacterial, antiviral, and antiparasitic activity^10–12^. By modulating the ERK-p65 signaling pathway, CTZ inhibits hepatocellular carcinoma cell migration and invasion^13^. CTZ induced the repolarization of macrophages and exhibited anticancer activity in a murine model of melanoma^14^. Furthermore, CTZ inhibits the proliferation of breast, oral, and endometrial cancer cells^15–17^. However, the effects of CTZ in MM have not been reported in previous studies.

Research has suggested the potential of CTZ as a treatment for MM. One study examined the effect of clotrimazole on the migration and invasion capabilities of the U266 cell line, suggesting its potential as a treatment for multiple myeloma. However, this report was limited to a single cell line and focused on tumor cell migration and invasion^18^. Another study discussed the clinical efficacy of bisphosphonates in reducing fracture risk in patients with multiple myeloma or osteoporosis and determined that antifungal drugs such as clotrimazole could be used if necessary, suggesting its potential safety for patients with MM^19^.

Drug repurposing expands the use of existing drugs, with comparatively lower development costs and shorter development time^20,21^. CTZ is a safe, old drug that has been in clinical use for many years, making it a good candidate for use in other diseases (Table 1). This study investigated the effect of CTZ on the proliferation of MM cells and the underlying mechanisms. The discovery sheds light on the mechanism of how CTZ inhibits cancer growth and underscores its promising potential for a novel approach to the treatment of MM and other malignancies.

**Table 1:** Overview of clotrimazole's antitumor effects in different types of cancer.

Author(s), year and refs	Cancer type	Cancer cell lines	Main findings	Mechanism of action	Dosage and administration	Conclusion
Liu et al. 2022^13^	Hepatocellular carcinoma	MHCC-97H, SMCC 7721, Hep3B, HuH7	Clotrimazole inhibits HCC cell migration and invasion	Inhibition of ERK phosphorylation and EMT	20 μM clotrimazole in HCC cells	Clotrimazole inhibits HCC metastasis through the suppression of EMT via the ERK pathway
Ochioni et al. 2021^14^	Melanoma	B16F10	Clotrimazole inhibits tumor growth, reduces lactate levels in the tumor microenvironment, and decreases vascular endothelial growth factor expression	Inhibition of the PI3K pathway, reduction of glycolysis, and repolarization of tumor-associated macrophages cells	200 mg/kg/day in vivo	Clotrimazole exerts anticancer effects by modulating the tumor microenvironment and TAM polarization
Bae et al. 2018^15^	Breast cancer	MCF-7, MDA-MB-231	Clotrimazole inhibited cell proliferation and invasiveness of breast cancer cells	Induction of apoptosis, G1 phase arrest, inhibition of MMP9	50 μM clotrimazole in breast cancer cells; 100 mg/kg/day in vivo	Clotrimazole shows strong antitumor activity. It has the potential to be repositioned as a cancer therapy
Wang et al. 2014^16^	Oral squamous cell carcinoma	CAL27, SCC25, UM1	Clotrimazole inhibited cell viability, colony formation, induced apoptosis, and reduced tumor growth	Induction of G0/G1 cell cycle arrest, down-regulation of Bcl-2, up-regulation of Bax	40 μM clotrimazole in vitro; 150 mg/kg/day in vivo	Clotrimazole shows potential as a therapeutic agent for OSCC by inducing apoptosis and inhibiting cell proliferation
Adinolfi et al. 2015^32^	Melanoma	A375	Clotrimazole induces cytotoxicity in A375 human melanoma cells, induces cell cycle arrest at the G1-S phase transition, and alters membrane-bound protein V responsiveness	Inhibits hexokinase, induces cell cycle arrest, induces apoptosis	10 μM Clotrimazole in A375 Cells	Clotrimazole has a pro-apoptotic effect on melanoma cells
Zuccolini et al. 2023^33^	Melanoma, pancreatic cancer	WM266-4, Panc-1	Clotrimazole causes a decrease in cell viability and migration in WM266-4 and Panc-1 cells, independent of IK expression levels	IK Independence effect	30 μM Clotrimazole in WM266-4 Cells	The effect of clotrimazole on cancer cells may be due to off-target effects on other cellular targets or blockade of IK channels located in intracellular organelles

*ERK* Extracellular signal-regulated kinase, *EMT* Epithelial–mesenchymal transition, *PI3K* Phosphoinositide 3-kinase, *TAM* Tumor-associated macrophages, *MMP* Matrix metalloproteinase, *IK* Inwardly rectifying potassium channels, *OSCC* Oral squamous cell carcinoma.

## Results

### CTZ inhibits MM cell proliferation

**Figure 1:**
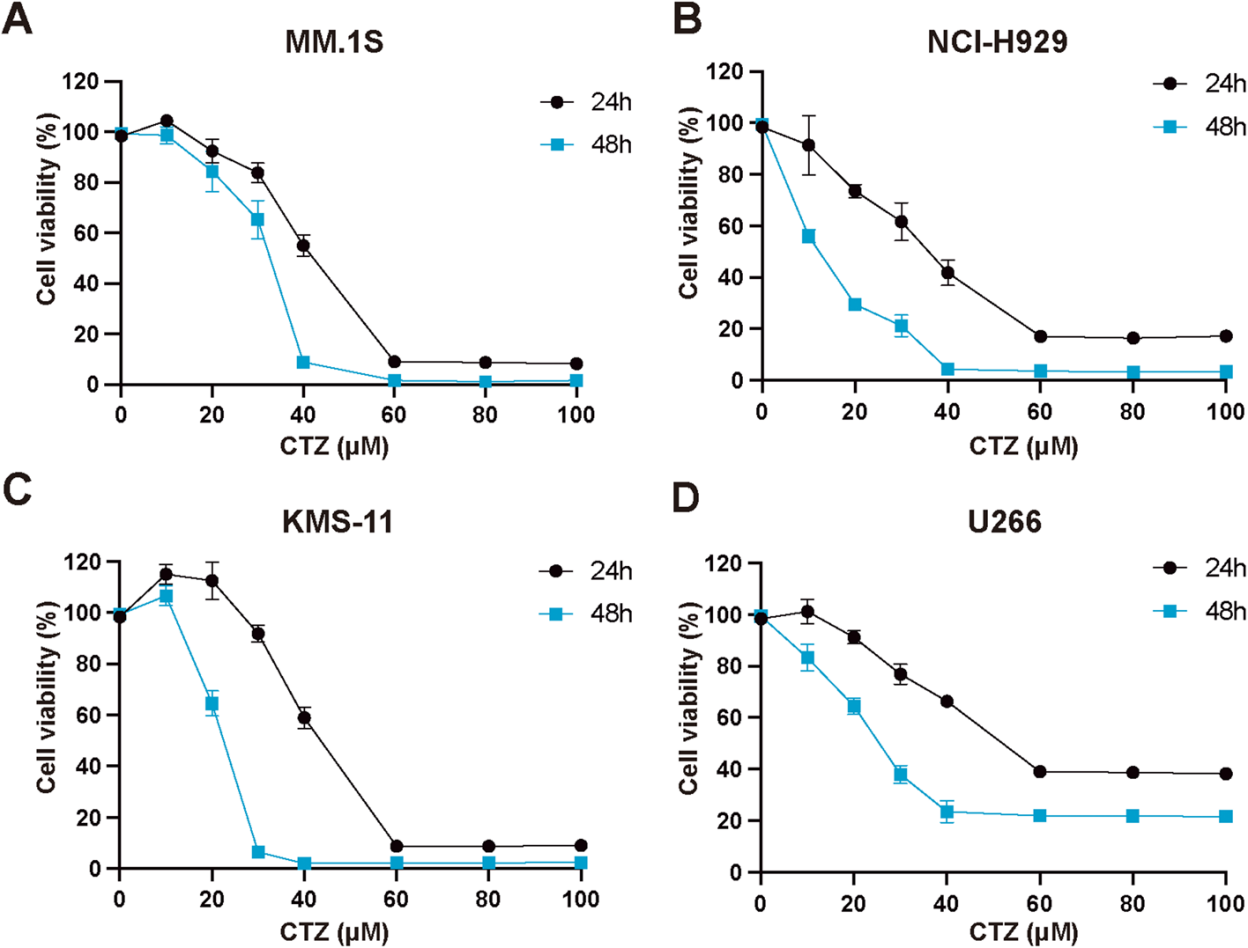
Impact of clotrimazole on the growth of multiple myeloma cells. Cells were cultured with clotrimazole for 24 or 48 h, using Cell Counting Kit-8 to evaluate cell viability. Growth curves of MM.1S (**a**), NCI-H929 (**b**), KMS-11 (**c**), and U266 (**d**) cells are shown. Each data point represents the mean ± standard deviation from three independent experiments conducted in triplicate.

CTZ decreased MM cell viability based on both dosage and time, exhibiting statistical significance (p ≤ 0.05) (Fig. 1). IC50 values in NCI-H929, MM.1S, KMS-11, U266 cells were 35.04 ± 1.63 μM, 40.05 ± 1.23 μM, 38.76 ± 1.3 μM, and 35.79 ± 1.91 μM after 24-h treatment and 18.25 ± 0.92 μM, 32.77 ± 0.35 μM, 20.51 ± 0.37 μM, and 23.85 ± 0.53 μM after 48-h treatment, respectively. These findings demonstrate that CTZ inhibits MM cell proliferation.

### CTZ induces G0/G1 cell cycle arrest in MM.1S and NCI-H929 cells

**Figure 2:**
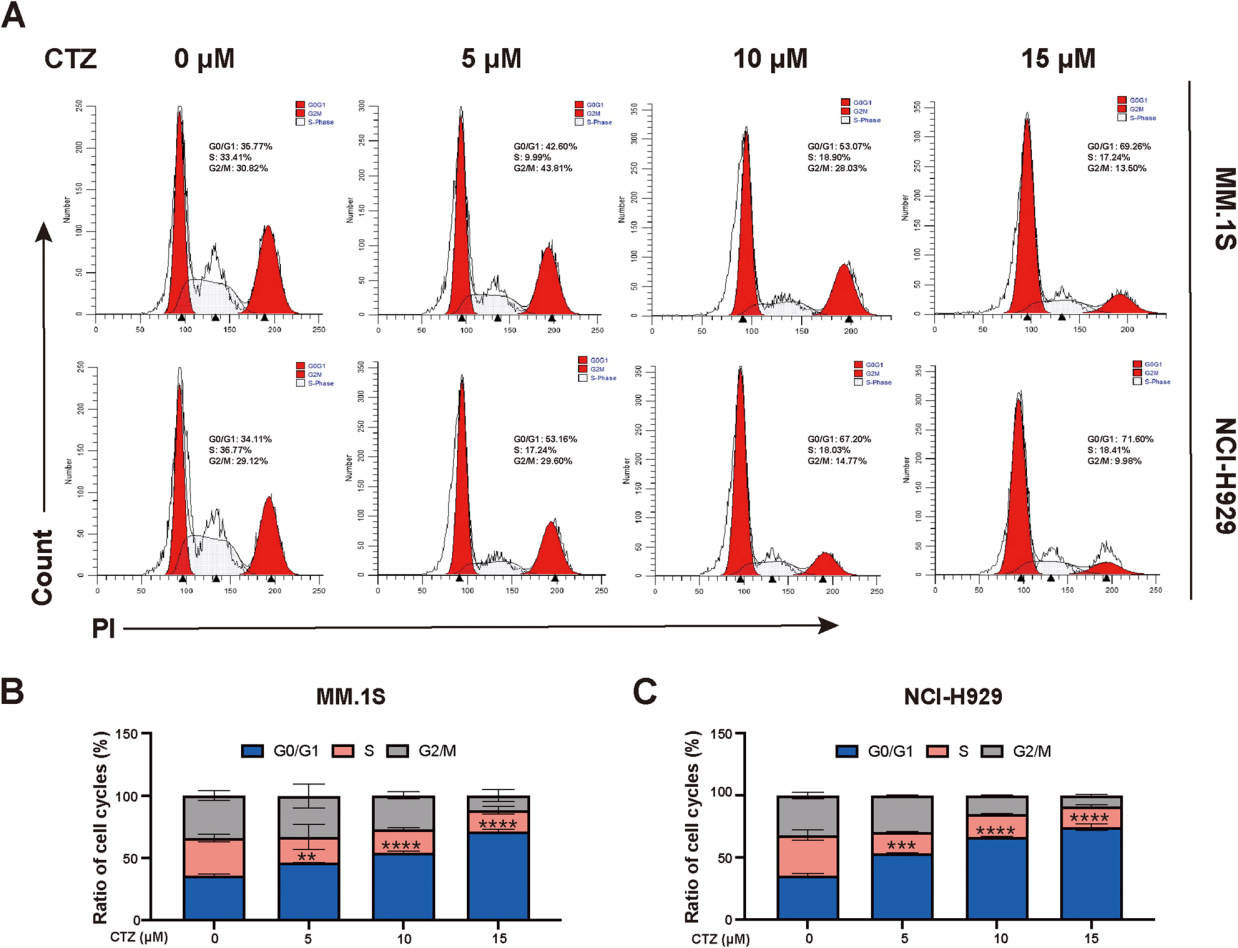
Effect of clotrimazole on the cell cycle of MM.1S and NCI-H929 multiple myeloma cells. (**a**) Cell cycle progression of MM.1S and NCI-H929 cells cultured with clotrimazole (0, 5, 10, 15 μM) for 12 h. (**b**,**c**) Cell cycle progression of MM.1S and NCI-H929 cells. *p < 0.05, **p < 0.01, ***p < 0.001, ****p < 0.0001 versus controls. *ns* not significant (p ≥ 0.05).

The percentage of MM.1S cells in the G0/G1 phase following 24-h treatment with 0, 5, 10, and 15 μM CTZ was 35.77%, 42.60%, 53.07%, and 69.26%, respectively. The proportion of NCI-H929 cells in the G0/G1 phase after 24-h exposure to these concentrations was 34.11%, 53.16%, 67.20%, and 71.60%, respectively (Fig. 2). These results indicate that CTZ induces G0/G1 arrest.

### CTZ induces apoptosis in MM.1S and NCI-H929 cells

**Figure 3:**
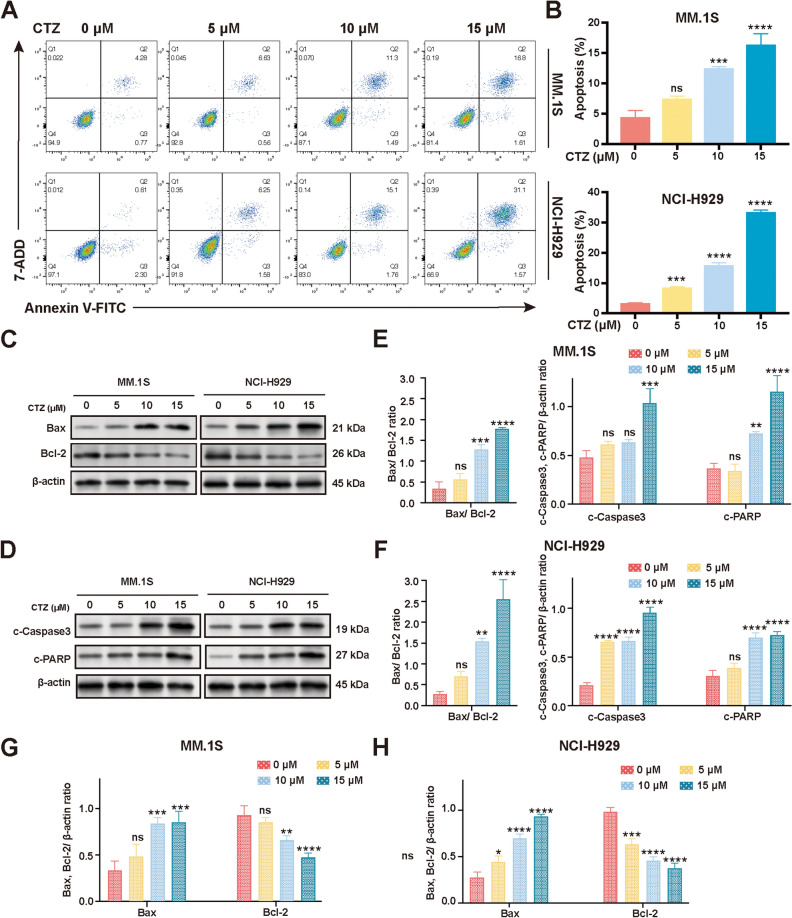
Effect of clotrimazole (CTZ) on inducing apoptosis in MM.1S and NCI-H929 multiple myeloma cells. (**a**) Flow cytometric analysis of the apoptosis rate in CTZ-treated cells stained with Annexin V-FITC/7-ADD. (**b**) Rate of apoptosis in CTZ-treated cells. (**c**) Western blot analysis of the expression of Bax and Bcl-2 in CTZ-treated cells. (**d**) Western blot analysis of the expression of cleaved caspase-3 and cleaved PARP in CTZ-treated cells. (**e**,**f**) Bax/Bcl-2 ratios and semiquantitative analyses of the relative protein expression of cleaved caspase-3 and cleaved PARP in MM.1S and NCI-H929 cells. (**g**,**h**) Western blot analysis of the expression of Bax and Bcl-2 in CTZ-treated MM.1S and NCI-H929 cells. The data are expressed as the mean ± standard deviation of three independent sets of assays conducted in triplicate. *p < 0.05, **p < 0.01, ***p < 0.001, ****p < 0.0001 versus controls. *ns* not significant (p ≥ 0.05).

CTZ dose-dependently increased the proportion of both the early and late apoptotic cells (Fig. 3a,b), demonstrating that CTZ induces apoptosis in MM cells.

### CTZ regulates expression of apoptosis proteins in MM.1S and NCI-H929 cells

Apoptosis, triggered by drugs, is a critical mechanism for cancer cell extinction^22^, and Bax and Bcl-2 are key mediators of apoptosis^23^. Western blot analysis demonstrated that CTZ dose-dependently raised the levels of the pro-apoptotic protein Bax and lowered the levels of the anti-apoptotic protein Bcl-2 in MM.1S and NCI-H929 cells, leading to an increased Bax/Bcl-2 ratio (with a maximal increase of 6.0-fold in MM.1S cells and 9.3-fold in NCI-H929 cells) compared to the control (Fig. 3c,e–h). Statistical analysis confirmed that these changes were significant. The results showed that CTZ decreased the protein expression of Bcl-2 (with a maximal decrease of 0.5-fold in MM.1S cells and 0.4-fold in NCI-H929 cells) while increasing the protein expression of Bax (with a maximal increase of 3.0-fold in MM.1S cells and 3.5-fold in NCI-H929 cells) compared to the control. PARP, an enzyme involved in DNA repair, is cleaved by caspases into cleaved PARP, which is inactive^24^. CTZ dose-dependently increased cleaved PARP as well as cleaved caspase-3 protein levels in both cell types in this study (Fig. 3d–f). Statistical analysis confirmed that these changes were significant. The results showed that elevated levels of cleaved caspase-3 (with an increase of 2.2-fold in MM.1S cells and 4.5-fold in NCI-H929 cells) and cleaved PARP (with an increase of 3.2-fold in MM.1S cells and 2.4-fold in NCI-H929 cells) were observed after 15 μM CTZ treatment compared to the control.

### CTZ increases total and mitochondrial ROS levels in NCI-H929 cells

**Figure 4:**
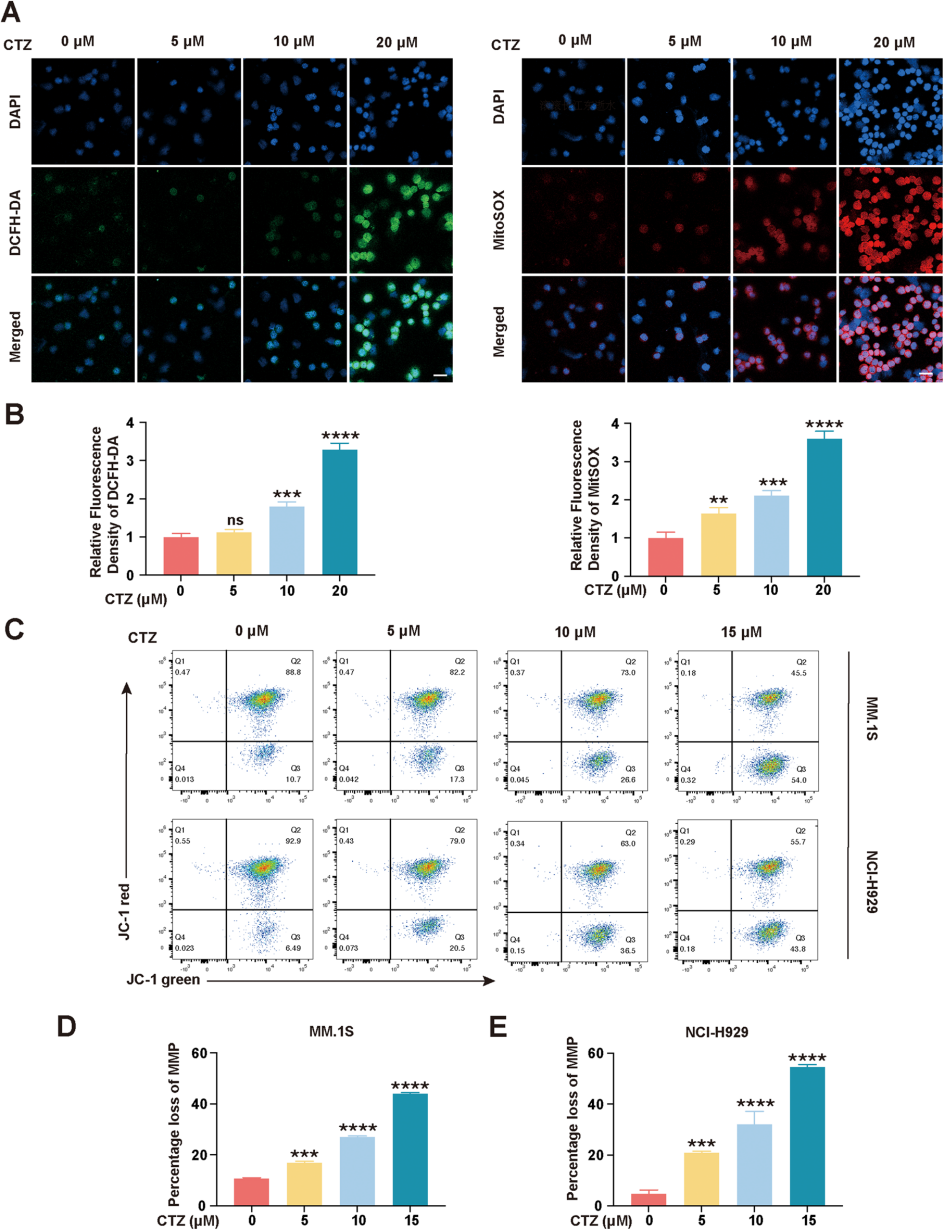
Alterations in reactive oxygen species (ROS) levels and mitochondrial membrane potential (MMP) by CTZ in multiple myeloma cells. (**a**) Fluorescence microscopy analysis of total ROS and mitochondrial ROS production in NCI-H929 cells treated with clotrimazole (CTZ). Scale bar: 10 μm. (**b**) Quantification of Fluorescence Intensity of DCFH-DA and MitoSOX in NCI-H929 cells treated with different concentrations of CTZ. (**c**) Using flow cytometry, MMP in MM.1S and NCI-H929 cells were cultured by different concentrations of CTZ and stained with JC-1 dye. (**d**,**e**) Rate of MMP loss in CTZ-treated cells. *p < 0.05, **p < 0.01, ***p < 0.001, ****p < 0.0001 versus controls. *ns* not significant (p ≥ 0.05).

Mitochondria generate ROS^25^. Excessive ROS induce oxidative damage, leading to mitochondrial events, including apoptosis. In this study, CTZ dose-dependently increased the fluorescence intensity of DCFH-DA and MitoSOX, demonstrating that CTZ raised both the total ROS and mitochondrial ROS at levels in NCI-H929 cells (Fig. 4a). Quantification of fluorescence intensity showed a significant increase in ROS levels with higher doses of CTZ, indicating that CTZ effectively induces oxidative stress in these cells (Fig. 4b).

### CTZ alters MMP in MM.1S and NCI-H929 cells

The mitochondrial pathway is associated with programmed cell death^26^. In the process of mitochondrial respiration and oxidation, the energy generated is stored in the inner mitochondrial membrane as electrochemical potential energy, creating an asymmetric distribution of protons and other ions on either side of the membrane to form the MMP^27^. A decrease in MMP is a hallmark feature of the early phases of apoptosis^28^. The potential involvement of the mitochondrial pathway in CTZ-induced cell death in MM.1S and NCI-H929 cells was investigated using JC-1 staining and FCM. We observed a dose-dependent decrease in MMP with increasing concentrations of CTZ. Specifically, treatment with 5, 10, and 15 μM CTZ resulted in a significant reduction of MMP in both MM.1S and NCI-H929 cells. The percentage loss of MMP was significantly elevated at the 15 μM dose, indicating a strong dose-dependent effect of CTZ on mitochondrial dysfunction (Fig. 4c–e).

### CTZ induces apoptosis in MM.1S and NCI-H929 cells by inhibiting the NF-κB signaling pathway

**Figure 5:**
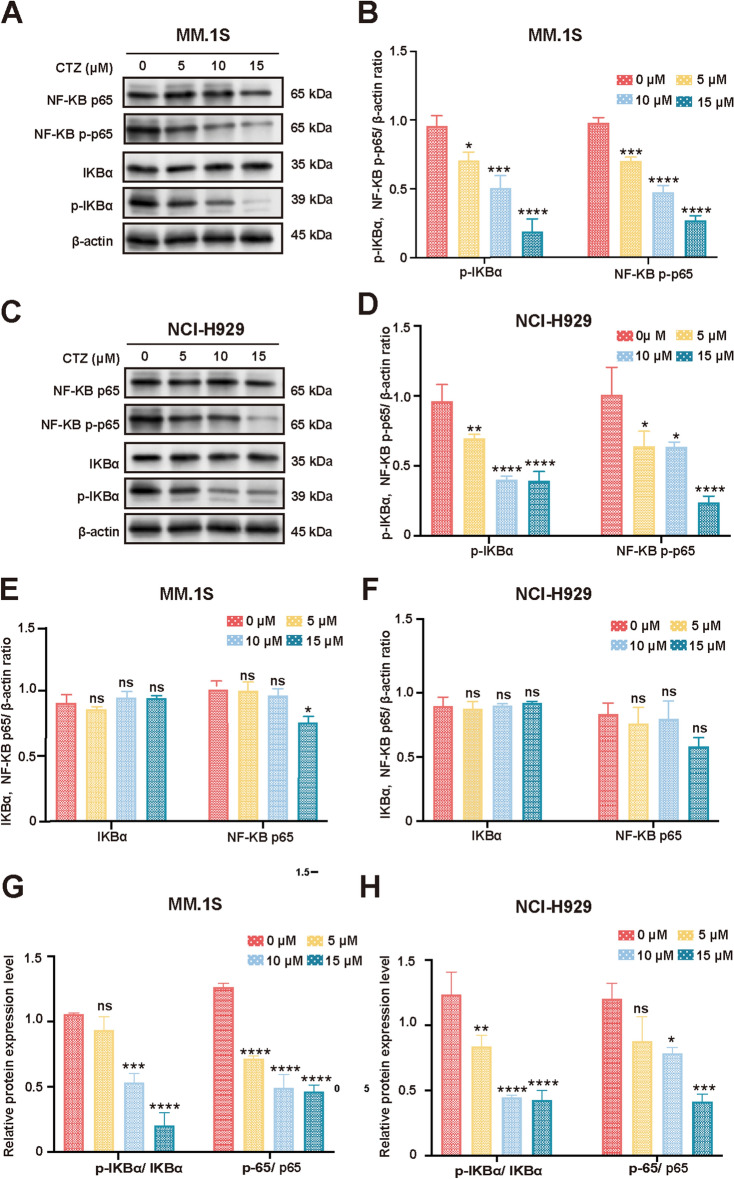
Regulation of NF-κB signaling pathway by CTZ in MM.1S and NCI-H929 cells. (**a**,**c**) Western blot analysis was performed to evaluate the levels of NF-κB pathway signaling components, including p65, p-p65, IκBα, and p-IκBα in MM.1S and NCI-H929 cells treated with CTZ. (**b**,**d**,**e**,**f**) Semiquantitative analysis of Western blot results showing the expression levels of p65, p-p65, IkBa, and p-IκBα in MM.1S and NCI-H929 cells after CTZ treatment. (**g**,**h**) Quantitation of the p-IκBα/IκBα and p-p65/p65 ratios in MM.1S and NCI-H929 cells treated with different concentrations of CTZ. The data are presented as the mean ± standard deviation from three independent experiments conducted in triplicate. *p < 0.05, **p < 0.01, ***p < 0.001, ****p < 0.0001. *ns* not significant (p ≥ 0.05).

The NF-KB pathway is a classic pathway in the development of MM. Next, we determined whether CTZ regulates the NF-κB pathway in MM.1S and NCI-H929 cells by measuring the protein expression of IκBα, phosphorylated IκBα, p65, and phosphorylated p65 (Fig. 5a,c). CTZ dose-dependently decreased the levels of phosphorylated p65 (with a maximal decrease of 0.3-fold in MM.1S cells and 0.2-fold in NCI-H929 cells) and phosphorylated IκBα (with a maximal increase of 0.3-fold in MM.1S cells and 0.4-fold in NCI-H929 cells) compared with the control, suggesting its inhibition of the NF-κB pathway in MM cells (Fig. 5b,d). Statistical analysis showed that these decreases were significant.

In addition, the protein level of IκBα did not change in MM.1S and NCI-H929 cells. For the expression level of p65 protein, there was no change in the 0–10 μM CTZ treated group in both cell lines. In 15 μM CTZ-treated MM.1S cells, the level of p65 was significantly reduced (to 0.7-fold of the control) with a p-value of less than 0.05, while in 15 μM CTZ-treated NCI-H929 cells, there was no statistically significant difference in the level of p65, although it was also reduced (to 0.8-fold of the control) (Fig. 5e,f). The results showed that CTZ decreased the p-IκBα/IκBα ratio (with a maximal decrease of 0.2-fold in MM.1S cells and 0.4-fold in NCI-H929 cells) and the p-p65/p65 ratio (with a maximal decrease of 0.4-fold in MM.1S cells and 0.3-fold in NCI-H929 cells) at the highest concentration (Fig. 5g,h). Significant reductions were observed at 10 μM and 15 μM CTZ (*p* < 0.001), indicating CTZ inhibits NF-κB pathway activity in both cell lines. These results indicate that CTZ effectively inhibits the phosphorylation of key proteins in the NF-κB pathway.

## Discussion

Over the past few years, the clinical use of proteasome inhibitors and immuno-modulators has significantly enhanced the prognosis of patients with MM^29^. Nevertheless, cases of relapsed and refractory MM are common. Moreover, the time to remission in patients with relapsed MM shortens as treatment progresses^30^. Therefore, MM is incurable, underscoring the need to develop effective therapeutic agents with few side effects. Drug repurposing in cancer is more cost-effective than developing new drugs^31^.

CTZ is a synthetic azole antifungal drug commonly used topically to treat tinea pedis, vulvovaginal, and oropharyngeal candidiasis^8^. It inhibits fungal growth by targeting ergosterol biosynthesis and is safe and well-tolerated with few adverse effects. Studies have shown that CTZ inhibits the proliferation of cancer cells through multiple mechanisms. For instance, Liu et al. reported that CTZ suppresses hepatocellular carcinoma cell migration and invasion by inhibiting ERK phosphorylation and epithelial-mesenchymal transition^13^. In breast cancer cell lines (MCF-7 and MDA-MB-231), Bae et al. observed that CTZ induces apoptosis and G1 phase arrest while inhibiting MMP9, thus reducing cell proliferation and invasiveness^15^. Further research by Wang et al. demonstrated that CTZ significantly reduces cell viability, colony formation, and tumor growth in oral squamous cell carcinoma both in vitro and in vivo, primarily through G0/G1 cell cycle arrest and modulation of apoptosis-related proteins^16^. In melanoma models, Ochioni et al. and Adinolfi et al. found that CTZ not only inhibits tumor growth but also modulates the tumor microenvironment by repolarizing tumor-associated macrophages and inducing apoptosis through hexokinase inhibition^14,32^. Additionally, Zuccolini et al. highlighted the potential off-target effects of CTZ on IK channels, affecting the viability and migration of melanoma and pancreatic cancer cells^33^. As evidenced by CCK-8 assays, CTZ suppresses the proliferation of MM cells in a concentration- and time-dependent manner.

Cancer is a condition characterized by uncontrolled cell growth due to dysregulation of the normal cell cycle, which affects the speed of cell proliferation^34^. The duration of the cell phase differs between cell types and is largely influenced by the duration of the G1 phase. Earlier research has demonstrated that CTZ causes G0/G1 cell cycle arrest in A375 human melanoma cells^14^ and oral squamous carcinoma cells^16^. We found that CTZ induced G0/G1 arrest in MM.1S and NCI-H929 cells, leading us to hypothesize that CTZ induces apoptosis in MM. The difference in cell cycle states observed between MM.1S and NCI-H929 cells could be attributed to the intrinsic variations in their cellular machinery and signaling pathways. For instance, differences in the expression levels of cyclins, cyclin-dependent kinases (CDKs), and CDK inhibitors could affect the sensitivity of these cells to CTZ-induced G0/G1 arrest. Additionally, the presence of mutations or differential activation of upstream regulatory pathways, such as the p53 and Rb pathways, may also contribute to the observed differences in cell cycle arrest.

Apoptosis maintains physiological homeostasis^35^ and eliminates cancer cells in response to external stimuli, such as small-molecule drugs^36,37^. Apoptosis involves decreased MMP and caspase-3 activation^38^. The externalization of PS was evaluated via annexin V-FITC/7-AAD staining. Moreover, the translocation of PS to the outer membrane is stimulated by apoptosis. Annexin V possesses a great binding capacity for PS. The effects of CTZ on apoptosis in MM cells were assessed using annexin V-FITC/7-AAD staining combined with flow cytometry. We found that 12-h treatment with 15 μM CTZ increased the apoptosis rate of MM.1S and NCI-H929 cells.

Apoptotic pathways can be either extrinsic or intrinsic^39^. Upon stimulation, Bcl-2 family proteins activate MMPs, which leads to the liberation of cytochrome c from the mitochondria, initiation of the caspase cascade, and eventual cell death^40,41^. CTZ induces apoptosis via Bcl-2 family proteins. Similarly, in oral squamous cell carcinoma, CTZ induces apoptosis by upregulating Bax and downregulating Bcl-2^16^. We found that CTZ suppressed Bcl-2 expression, raised Bax levels, and triggered cleavage of PARP and caspase-3, indicating CTZ's engagement in the intrinsic apoptotic pathway. The significant increase in Bax and decrease in Bcl-2 levels in MM.1S and NCI-H929 cells treated with 15 μM CTZ (p-values < 0.01) underscores the role of CTZ in driving apoptosis via the intrinsic pathway. Elevated levels of cleaved caspase-3 and cleaved PARP in both cell lines (p-values < 0.001) further affirm this mechanism. Cleaved caspase-3, a principal executor of apoptosis, and cleaved PARP, a substrate of caspase-3, are essential indicators of apoptosis^42^. Their heightened levels signify the activation of the intrinsic apoptotic pathway, substantiating the involvement of CTZ in apoptosis induction.

Oxidative stress has an essential role in cancer therapy^43^. Excessive ROS causes mitochondrial membrane permeabilization, ultimately triggering apoptosis^44^. Previous studies have revealed that CTZ inhibits the growth of Leishmania parasites by impairing mitochondrial function and increasing ROS production^12^. CTZ also increases ROS production in fungal cells^45^. However, little information is available on the impact of CTZ on ROS production in MM cells. In this study, we evaluated overall and mitochondrial levels of ROS using DCFH-DA and MitoSOX staining. CTZ dose-dependently increased total and mitochondrial ROS generation while reducing MMP in MM.1S and NCI-H929 cells.

NF-κB consists of a dimerization complex involving either p50 (NF-κB1) or p52 (NF-κB2), along with various members of the Rel family (P65, c-Rel, and Rel-B) and functions as a transcription factor^46^. The NF-κB pathway is inactive in normal cells and activated in tumors, including MM^47–49^. IκB inhibits NF-κB, thereby inhibiting its movement to the nucleus. Upon phosphorylation, IκB releases NF-κB, which subsequently undergoes phosphorylation and translocation to the nucleus, inducing gene transcription^50^. In MM.1S and NCI-H929 cells, the dose-dependent reduction in phosphorylated p65 and phosphorylated IκBα (p < 0.0001) demonstrates that CTZ effectively inhibits the NF-κB pathway. Importantly, the protein level of IκBα remained unchanged, indicating that CTZ specifically targets the phosphorylation states rather than the overall protein levels of these components. Regarding the expression level of p65 protein, no alteration was observed in the 0–10 μM CTZ treated groups across both cell lines. In 15 μM CTZ-treated MM.1S cells, the level of p65 was significantly decreased (p-value < 0.05), whereas in 15 μM CTZ-treated NCI-H929 cells, a reduction in p65 levels was noted, although it did not reach statistical significance. These findings support that CTZ’s inhibition of the NF-κB pathway contributes to its apoptotic effects in MM cells.

The differential sensitivities of MM.1S and NCI-H929 cells to CTZ treatment might stem from their distinct regulatory mechanisms or cellular environments, meriting further exploration. Various cell lines may possess unique gene expression profiles and signalling pathways, potentially influencing the ratios of p-IkBα/IkBα and p-65 NF-κB/NF-κB. Factors such as growth stage, proliferation status, and environmental conditions could also result in variations in protein expression levels. Moreover, despite our efforts to maintain consistent experimental conditions, minor variations during the experimental process could impact protein expression levels.

This study also has some limitations. Our apoptosis assay did not include essential positive and negative controls, such as ZVAD and staurosporine, which are critical for validating the results. We intend to incorporate these controls in our future experiments to enhance the robustness of our findings. Additionally, human mononuclear cells (PBMC) were not included as a negative control in the CCK-8 study. We acknowledge that incorporating PBMC cells allows for a more comprehensive assessment of drug toxicity. We will refine this experiment in our subsequent studies. Furthermore, our study did not include in vivo experiments, which are critical for validating the clinical relevance of our findings. The effects of CTZ were only tested on a limited number of MM cell lines, which may not fully represent the diversity of MM in patients. Additionally, we did not evaluate the long-term effects of CTZ treatment or potential mechanisms of resistance that may develop with prolonged exposure. Future studies should include long-term assays and analyze resistance mechanisms. Another limitation is that we did not evaluate the potential synergistic effects of combining CTZ with other chemotherapeutic agents. Exploring combination therapies may improve treatment efficacy and reduce side effects. Finally, although CTZ is known to be safe for its antifungal use, its safety profile in cancer treatment, including optimal dosage and potential side effects, requires thorough preclinical evaluation.

In the future, we intend to conduct a series of in vivo animal studies to further explore the role of clotrimazole in multiple myeloma and to verify its efficacy and safety. Additionally, we will explore the possibility of combining clotrimazole with other chemotherapeutic agents to develop more effective treatment strategies through this comprehensive research approach. These studies will not only help validate the efficacy of clotrimazole but also offer new directions and ideas for clinical treatment.

In conclusion, CTZ inhibits MM by regulating the expression of critical apoptosis proteins, including cleaved caspase-3, cleaved PARP, Bcl2, and Bax, through increased ROS production, G0/G1 arrest, and NF-κB signaling inhibition. These findings demonstrate that CTZ has a high potential to treat MM and other cancers.

## Methods

### Materials

CTZ (Aladdin Biological Technology, Shanghai, China) was stored at − 80 °C after being resolved in dimethyl sulfoxide (Sigma, MO, USA). Cell Counting Kit-8 (CCK-8) was offered by Dojindo (Kumamoto, Japan). We acquired basal RPMI-1640 medium, penicillin–streptomycin solution, and fetal bovine serum (FBS) by Gibco (Grand Island, NE, USA). The fluorescein isothiocyanate (FITC)-annexin V Apoptosis Detection Kit, which includes 7-amino actinomycin D (7-AAD), was procured from BD Biosciences (Franklin Lakes, NJ, USA). Mitochondrial Membrane Potential Assay Kit (including JC-1), Cell Apoptosis and Cycle Assay Kit, DCFH-DA, DAPI, MitoSOX Red, poly-l-lysine, RIPA buffer, proteinase, and phosphatase inhibitor cocktail, and a high-sensitivity ECL chemiluminescence kit were sourced from Beyotime Biotechnology (Shanghai, China). A BCA protein assay kit was offered by Thermo Fisher Scientific (Waltham, MA, USA). Polyvinylidene fluoride (PVDF) membranes were supplied by Merck (Rowe, NJ, USA). Primary antibodies against cleaved caspase-3, Bax, Bcl-2, cleaved poly ADP-ribose polymerase (PARP), p65, IκBα, p-p65, p-IκBα, and β-actin were provided by CST (Boston, MA, USA). Secondary antibodies, anti-rabbit immunoglobulin (Ig)G and anti-mouse IgG, were obtained from Beyotime Biotechnology (Shanghai, China).

### Cell culture

The human MM cell lines (U266, NCI-H929, KMS-11, MM.1S) were supplied from the Chinese Academy of Medical Sciences (Beijing, China) and cryopreserved using liquid nitrogen. Cells were incubated in RPMI-1640 medium, which included 10% FBS and 1% penicillin–streptomycin. Cells were placed in a humidified incubator set at 37 °C with a CO_2_ concentration of 5%. Cells in the logarithmic growth phase were selected for subsequent assays.

### Cell viability assay

CCK-8 assays were conducted to measure the impact of CTZ on cell viability. MM cells were plated at a dose of 1.0 × 10^4^ cells/well in 96-well plates following exposure to different concentrations of CTZ (0, 10, 20, 30, 40, 60, 80, and 100 μM) for either 24 or 48 h. After incubation at 37 °C for 2 h, 10 µL of CCK-8 reagent was added to each microwell. The measurement of absorbance at 450 nm was performed utilizing a microplate reader (Multiskan SkyHigh; Thermo Fisher Scientific, Waltham, MA, USA). Cell viability was determined according to the equation [(As − Ab)/(Ac − Ab)] × 100%, in which As represents the absorbance in the test wells, Ac indicates the absorbance in the control wells, and Ab shows the absorbance in the blank wells. Dose–response curves were plotted using GraphPad Prism version 9.0, and IC50 values were calculated.

### Cell cycle analysis

MM.1S and NCI-H929 cells (5.0 × 10^5^ cells/well) were plated in 6-well dishes and incubated with CTZ at a dose of 0, 5, 10, or 15 μM for 12 h. After collection by centrifugation, they were resuspended in PBS, immobilized in 70% ethanol, and left overnight at 4 °C. Subsequently, cells were cultured for 30 min in the dark at 37 °C with a mixture consisting of buffer, RNase A, and propidium iodide as instructed by the manufacturer. Flow cytometry (FACS Canto II; BD Biosciences, Franklin Lakes, NJ, USA) was performed for the cell cycle. Data handling and statistical analysis were performed with Modfit software (version 6.0).

### Cell apoptosis assay

In 12-well plates, MM.1S and NCI-H929 cells were plated at a concentration of 1.0 × 10^5^ cells/well and exposed for 12 h to CTZ at concentrations of 0, 5, 10, or 15 μM. Five microliters of FITC-conjugated Annexin V and an additional 5 µL of 7-AAD were then inoculated into each well. Subsequently, the wells were kept at 25 °C for 15 min. Apoptosis was analyzed using FCM, and data were processed and analyzed using the FlowJo software (version 9).

### Evaluation of ROS production

To measure the total ROS production in NCI-H929 cells, cells were grown in 12-well dishes and exposed to 0, 5, 10, or 15 μM CTZ for 6 h. In the dark, Cells were dyed with DCFH-DA and DAPI at 37 °C for 30 min. Following that, cells were gathered by centrifugation, washed three times with serum-free medium, and seeded in confocal dishes (Thermo Fisher Scientific) coated with poly-l-lysine for 6 h. Cells were imaged on a confocal microscope (Olympus FV3000, Tokyo, Japan).

To measure mitochondrial ROS production, NCI-H929 cells were plated in 12-well dishes and exposed to CTZ at concentrations of 0, 5, 10, or 15 μM for 6 h. Cells were dyed with MitoSOX Red and DAPI at 37 °C for 10 min in the dark. MitoSOX produces red fluorescence upon reacting with ROS. After centrifugation, the cells were washed with Hank’s balanced salt solution to seed confocal dishes coated with poly-L-lysine. Cells were imaged on a confocal microscope (Olympus FV3000, Tokyo, Japan).

### MMP analysis

MM.1S and NCI-H929 cells in 6-well plates were seeded and exposed to treatment with CTZ (0, 5, 10, or 15 μM) for a duration of 12 h. Cells were collected by centrifugation, resuspended in RPMI-1640 basal medium, and dyed with JC-1 staining agent for 25 min at 37 °C in the dark. Cells were analyzed using FCM. When MMP is elevated, JC-1 accumulates within the mitochondrial matrix, resulting in the generation of red fluorescence. In cases of low MMP, JC-1 fails to aggregate within the mitochondrial matrix, resulting in the generation of green fluorescence.

### Western blot analysis

MM.1S and NCI-H929 cells were cultured with different concentrations of CTZ for 24 h, collected by centrifugation, and lysed utilizing a lysis solution comprising proteinase and phosphatase inhibitors. The lysate was placed on ice for 25 min before being spun at 12,000 rpm for 20 min at 4 °C. Protein quantification was conducted using a BCA protein assay kit. After separation using 12% sodium dodecyl sulfate–polyacrylamide gel electrophoresis, proteins were transferred onto PVDF membranes via electro-transference. These membranes underwent blocking with 5% skim milk for 2 h at 37 °C, followed by overnight exposure at 4 °C to primary antibodies directed against Bcl-2, Bax, cleaved PARP, cleaved caspase-3, nuclear factor (NF)-κB p65, NF-κB p-p65, IκBα, and p-IκBα. The membranes were rinsed with TBS and subsequently incubated with secondary antibodies (either goat anti-rabbit or goat anti-mouse) for 2 h at 25 °C. Immunoreactive bands were captured using a chemiluminescence imaging system (Tanon-4200, Shanghai, China). The integrated optical density of the signals was measured semi-quantitatively using ImageJ software (version 1.5.3).

### Statistical analyses

GraphPad Prism version 9.0 (GraphPad Software Inc., San Diego, CA, USA) was used to perform statistical analyses. Student’s t-test was used to determine the significance of the differences between the means. Differences between the multiple groups were evaluated using a one-way analysis of variance. Each experiment was repeated independently three times, with data reported using the mean ± standard deviation. A significance level of less than 0.05 for the p-value was regarded as an indication of statistical significance.

### Supplementary Information

Supplementary Information 1.

Supplementary Information 2.

Supplementary Information 3.

Supplementary Figure 1.

## Data Availability

The data provided is accessible in the article.
