# Supplementary material for: Clotrimazole inhibits growth of multiple myeloma cells in vitro via G0/G1 arrest and mitochondrial apoptosis
Source: Sci Rep. 2024 Jul 4;14:15406. doi: 10.1038/s41598-024-66367-5 (PMC11224322; doi:10.1038/s41598-024-66367-5)

Bax (1) 20 kDa

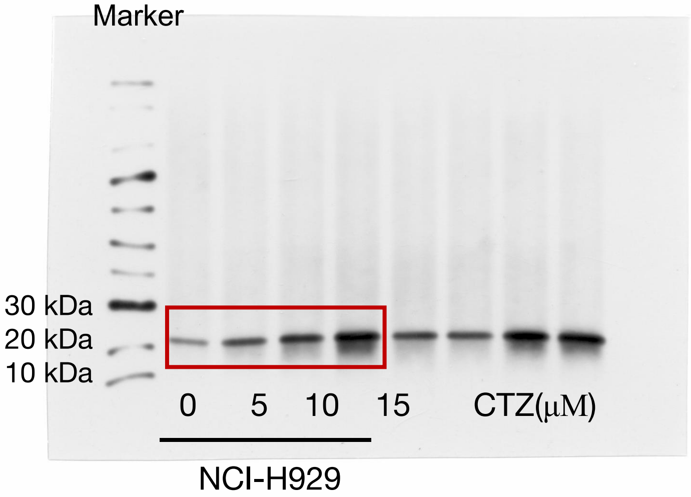

Bax (2) 20 kDa

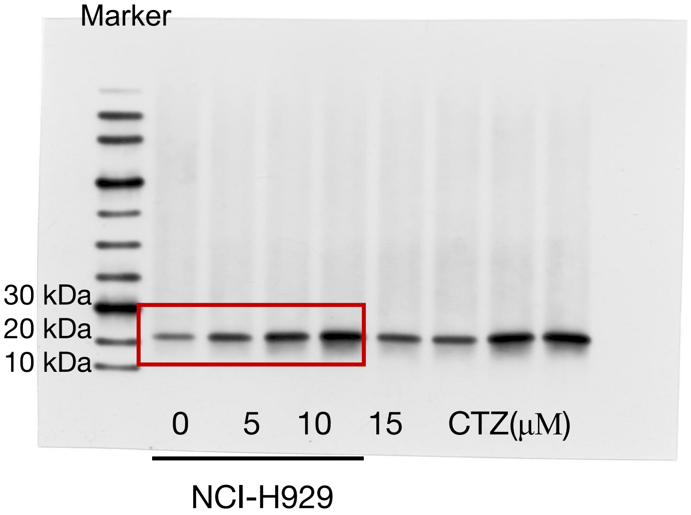

Bax (3) 20 kDa

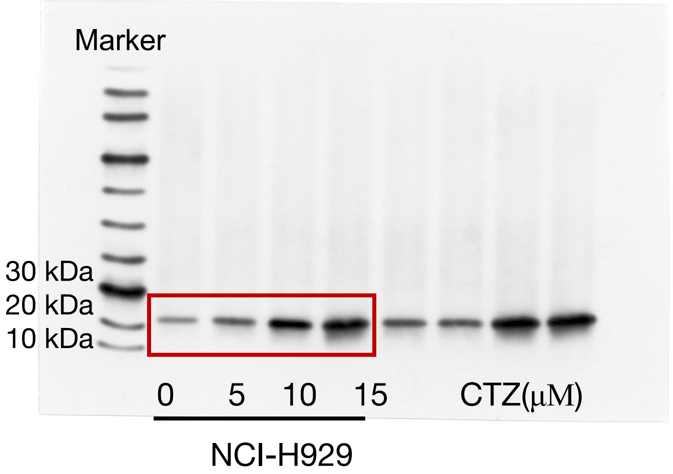

Bcl-2(1) 26KDa

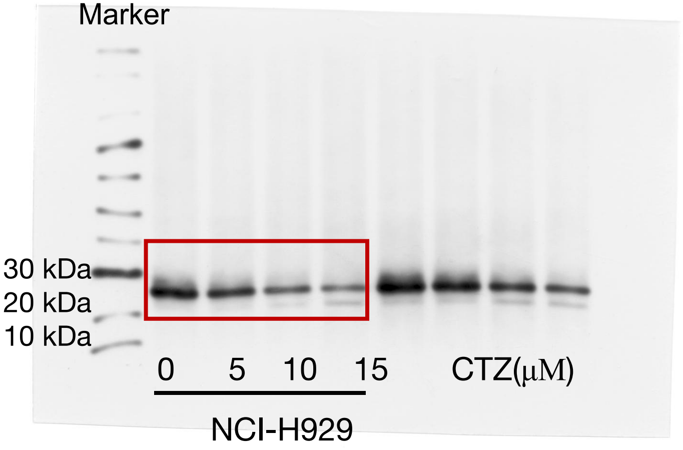

Bcl-2(2) 26KDa

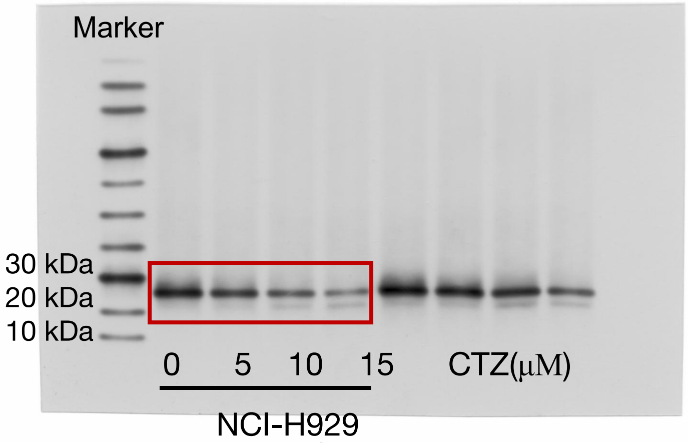

Bcl-2(3) 26KDa

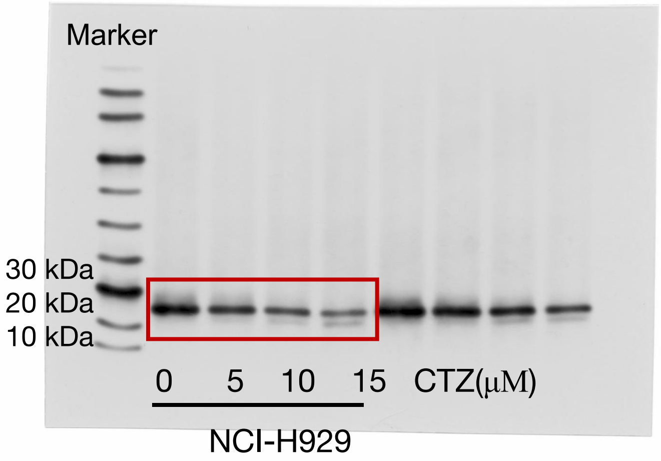

β-actin(1) 45kDa

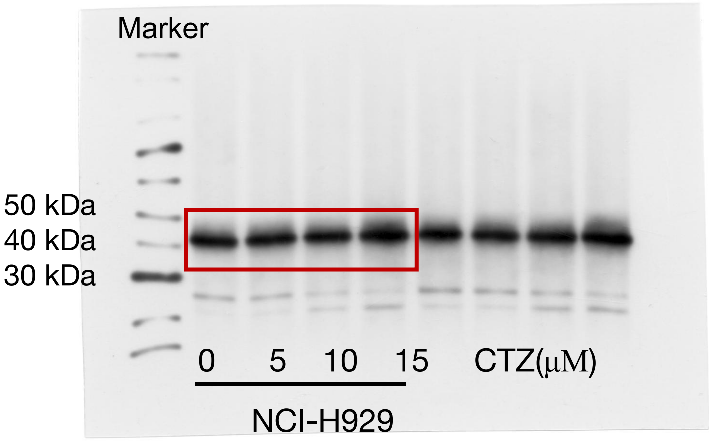

β-actin(2) 45kDa

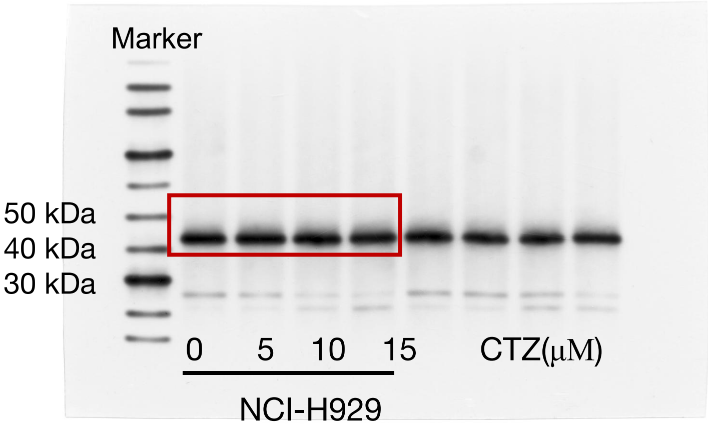

β-actin(3) 45kDa

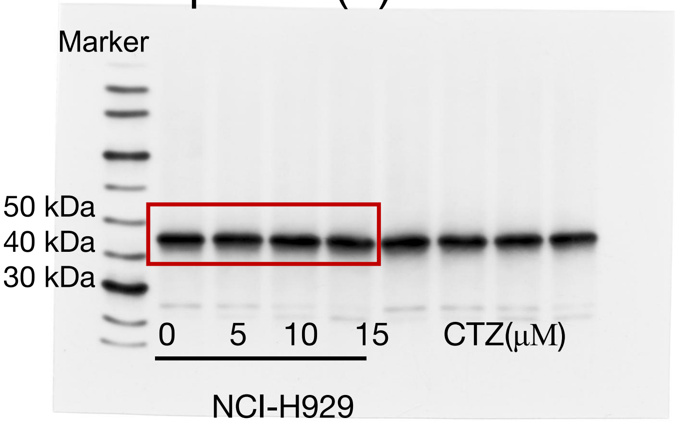

Bax (1) 20 kDa

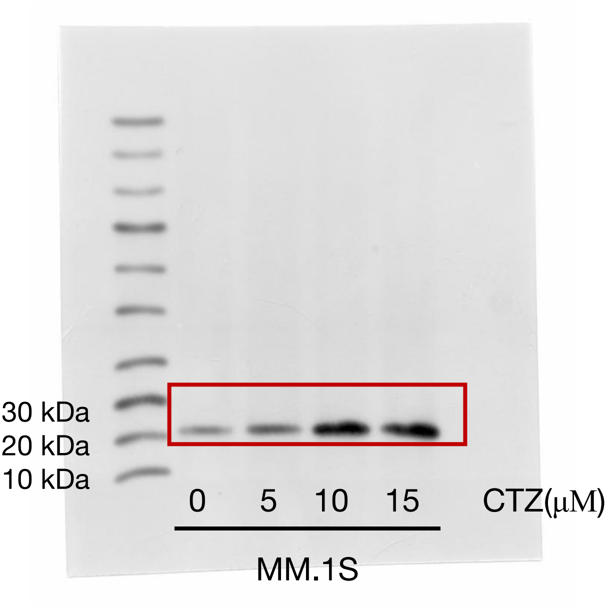

Bax (2) 20 kDa

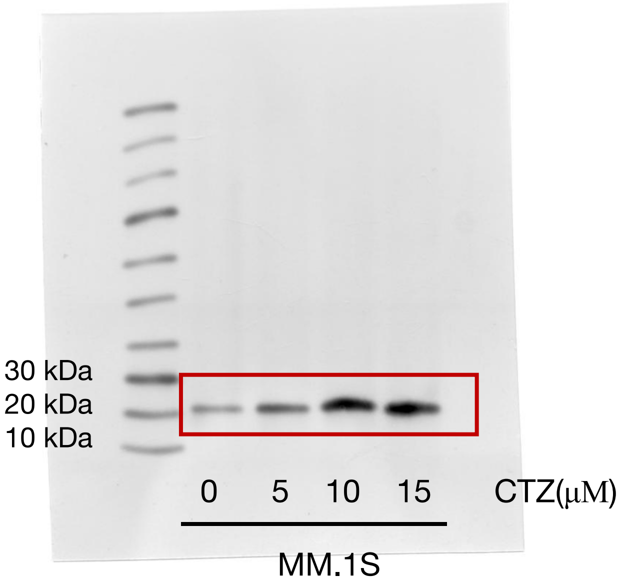

Bax (3) 20 kDa

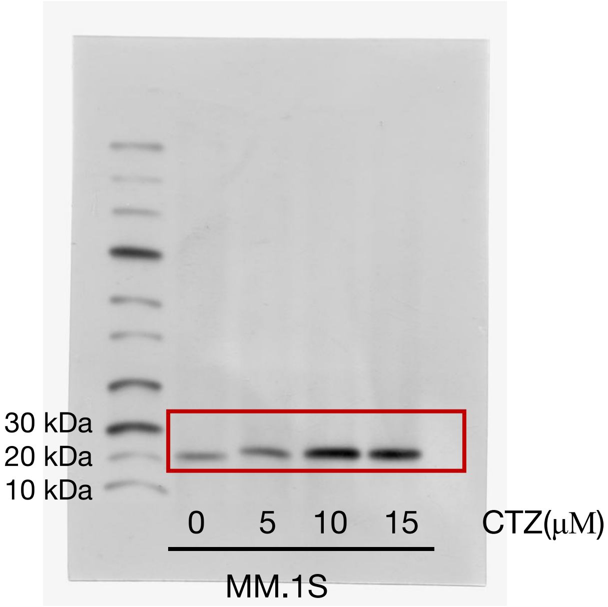

Bcl-2 (1) 20 kDa

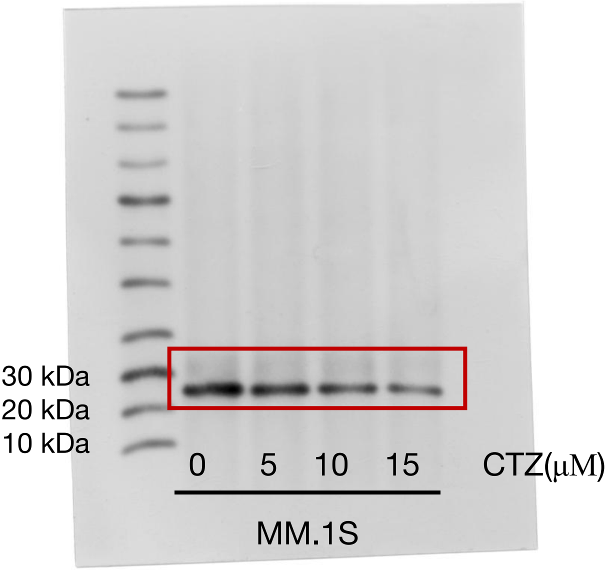

Bcl-2 (2) 20 kDa

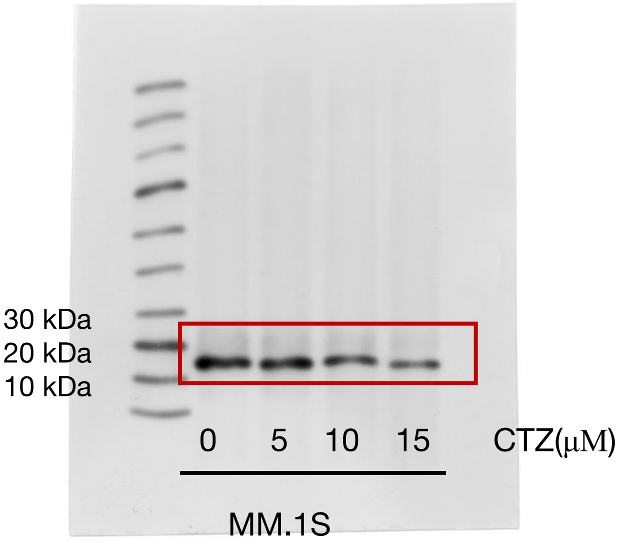

Bcl-2 (3) 20 kDa

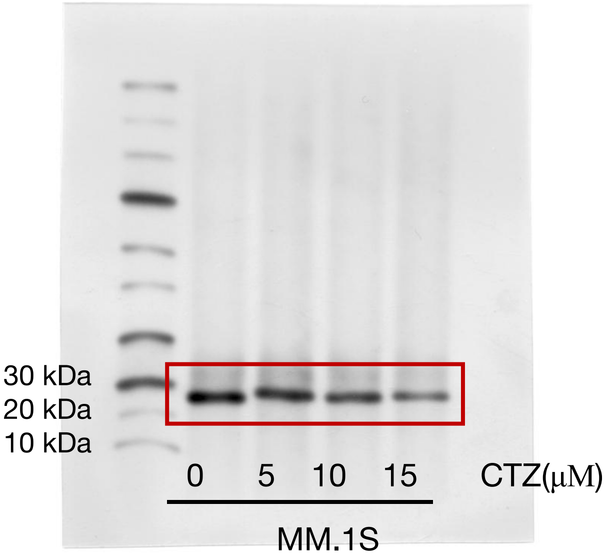

β-actin(1) 20 kDa

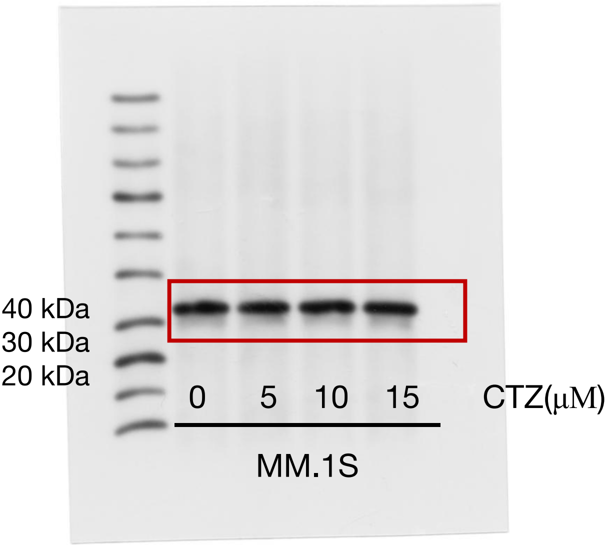

β-actin(2) 20 kDa

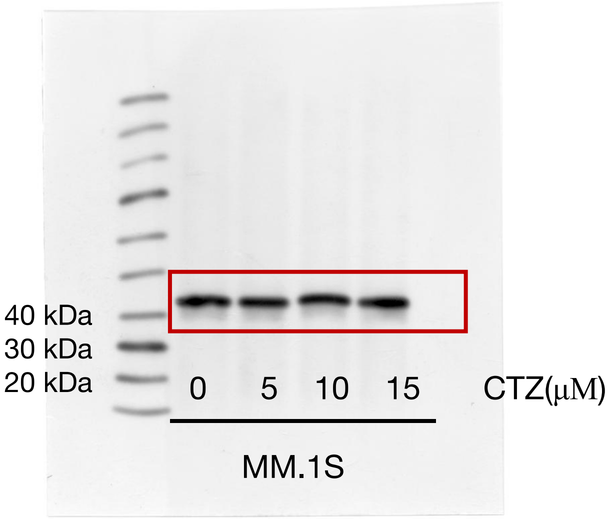

β-actin(3) 20 kDa

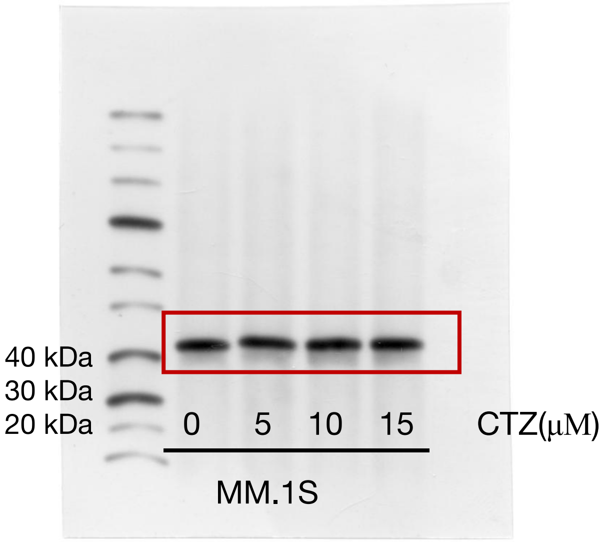

Supplement: Supplementary file 1 — Supplementary Information 1. [file 41598_2024_66367_MOESM1_ESM.pdf]
