# Supplementary material for: Clotrimazole inhibits growth of multiple myeloma cells in vitro via G0/G1 arrest and mitochondrial apoptosis
Source: Sci Rep. 2024 Jul 4;14:15406. doi: 10.1038/s41598-024-66367-5 (PMC11224322; doi:10.1038/s41598-024-66367-5)
Supplement: Supplementary file 2 — Supplementary Information 2. [file 41598_2024_66367_MOESM2_ESM.pdf]

Cleaved-caspase 3(1) 19kDa

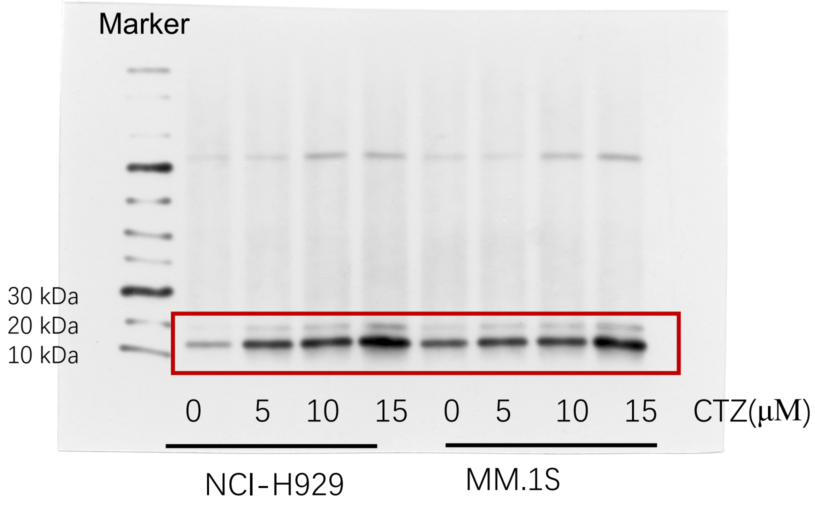

Cleaved-caspase 3(2) 19kDa

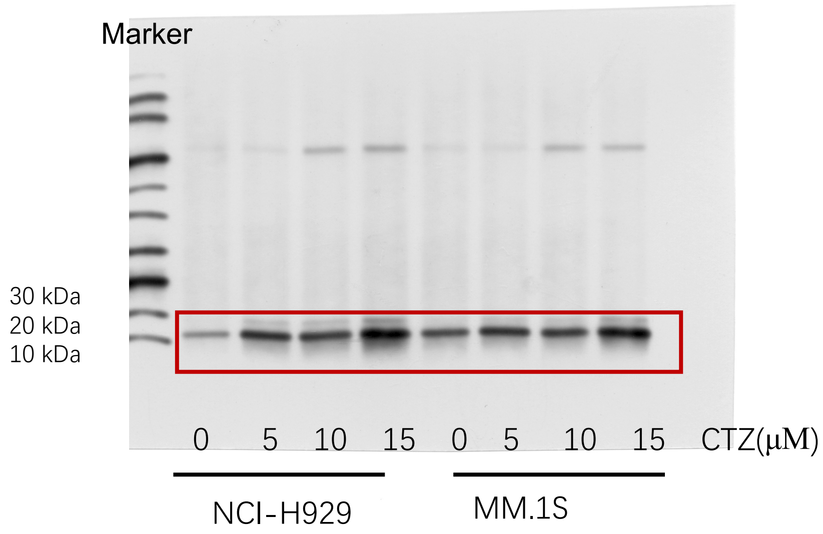

Cleaved-caspase 3(3) 19kDa

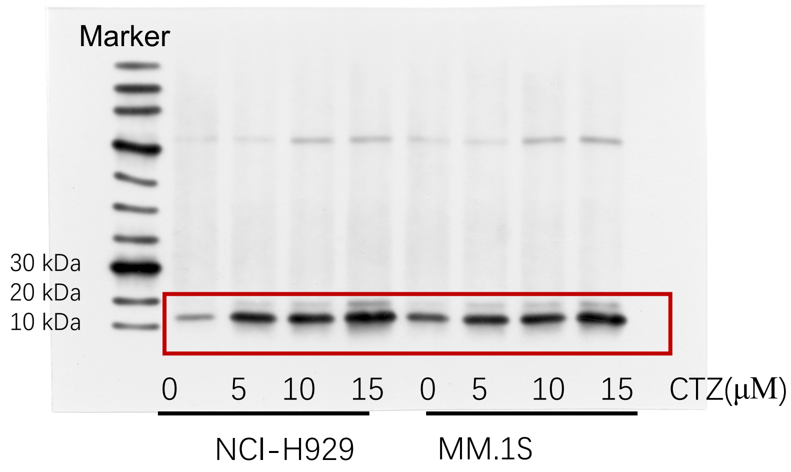

Cleaved-PARP(1) 89kDa

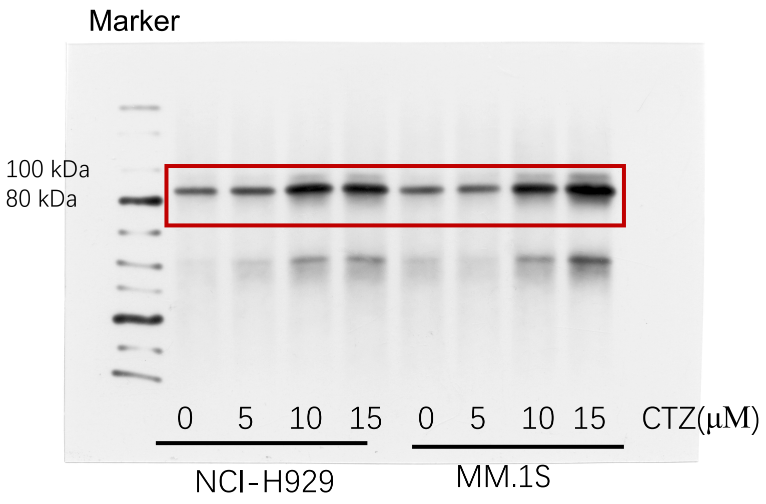

Cleaved-PARP(2) 89kDa

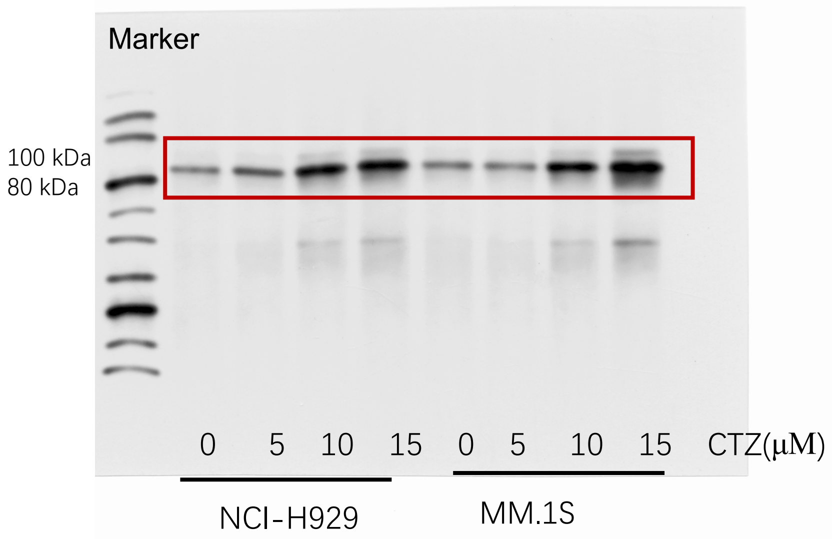

Cleaved-PARP(3) 89kDa

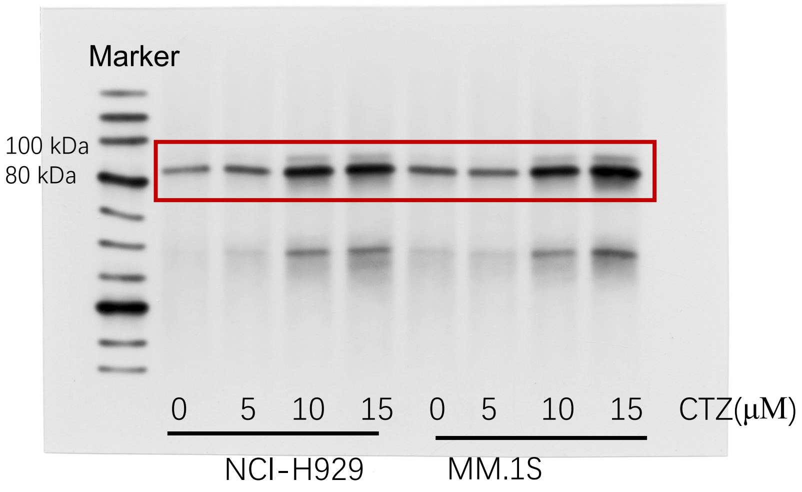

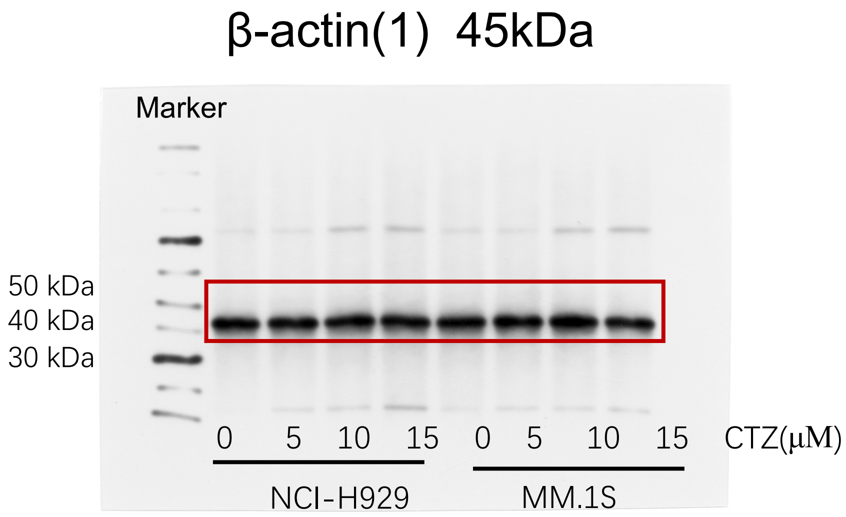
β-actin(1) 45kDa
Marker
50 kDa
40 kDa
30 kDa
0 5 10 15 0 5 10 15 CTZ(μM)
NCI-H929
MM.1S

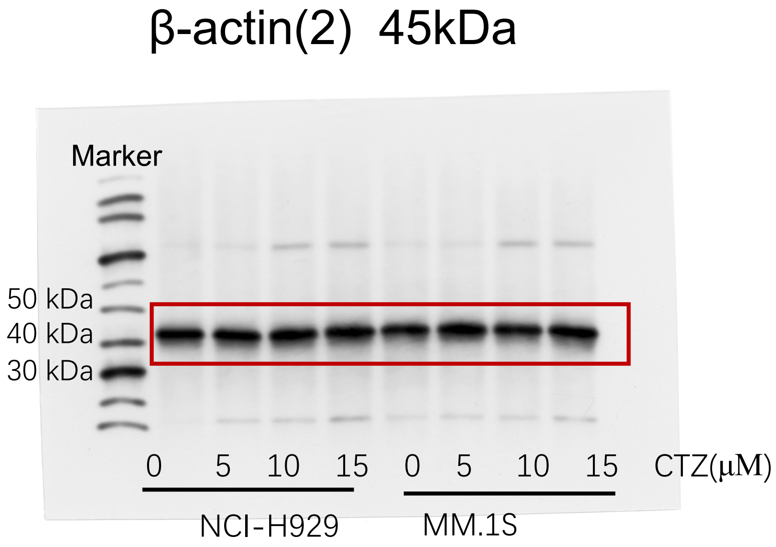
β-actin(2) 45kDa
Marker
50 kDa
40 kDa
30 kDa
0 5 10 15 0 5 10 15 CTZ(μM)
NCI-H929
MM.1S

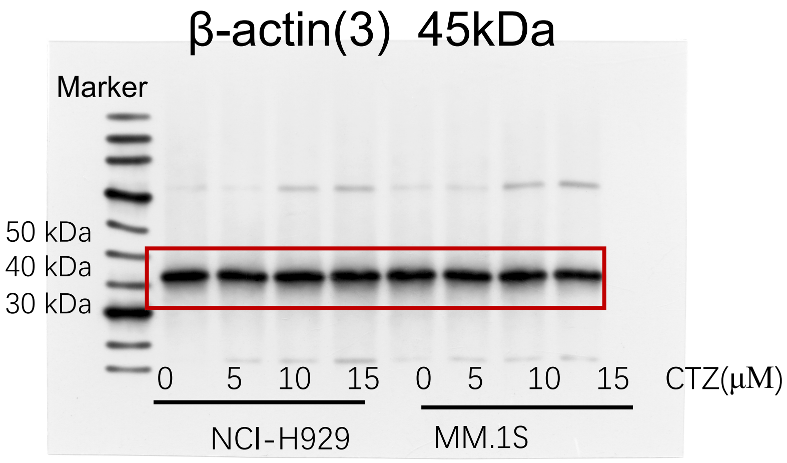
β-actin(3) 45kDa
Marker
50 kDa
40 kDa
30 kDa
0 5 10 15 0 5 10 15 CTZ(μM)
NCI-H929
MM.1S
