# Supplementary material for: Clotrimazole inhibits growth of multiple myeloma cells in vitro via G0/G1 arrest and mitochondrial apoptosis
Source: Sci Rep. 2024 Jul 4;14:15406. doi: 10.1038/s41598-024-66367-5 (PMC11224322; doi:10.1038/s41598-024-66367-5)

IκBα(1) 39kDa

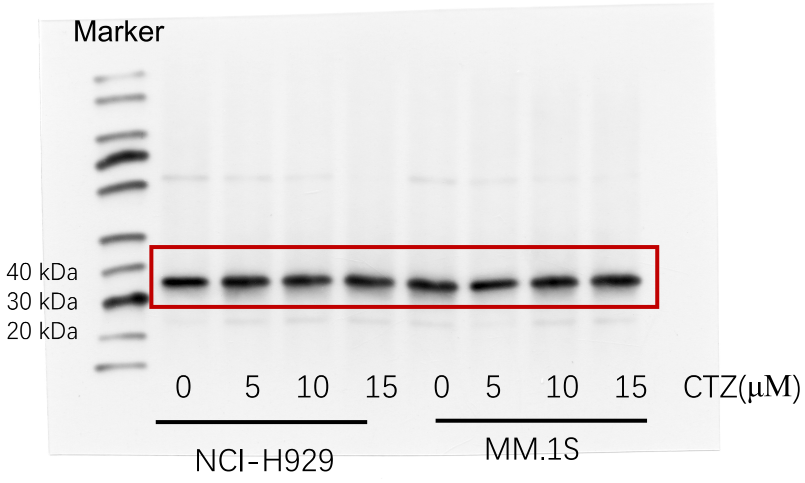

IκBα(2) 39kDa

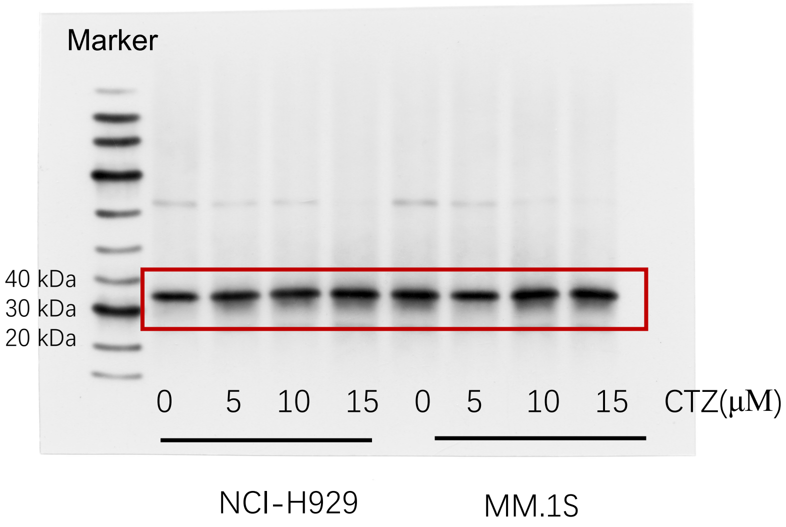

IκBα(3) 39kDa

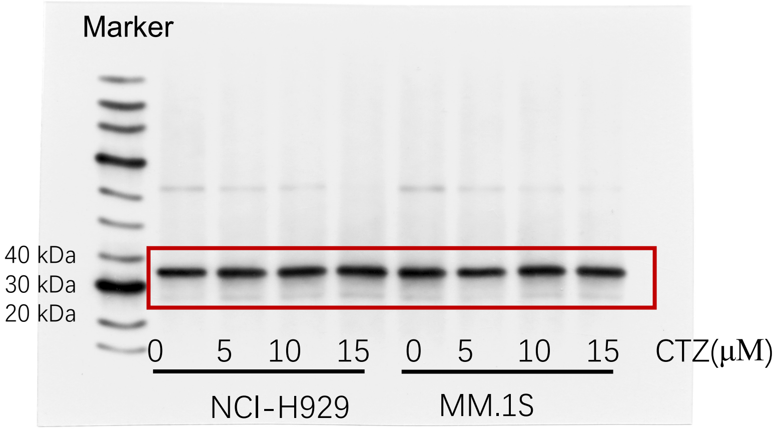

p-IκBα(1) 40kDa

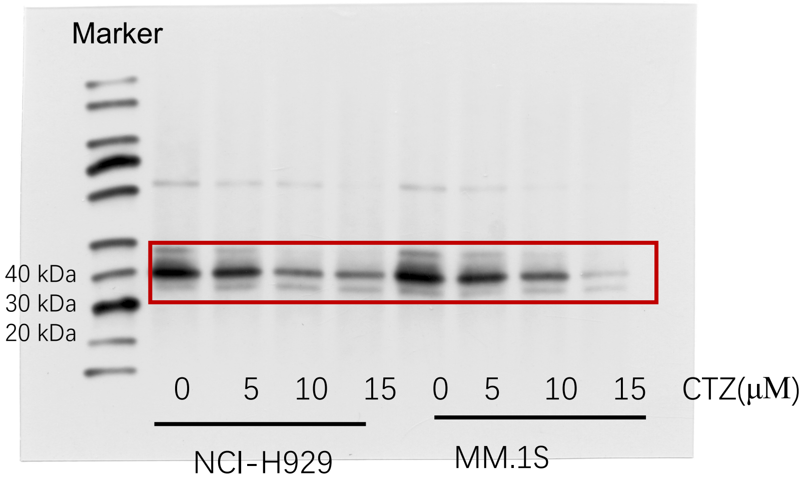

p-IκBα2) 40kDa

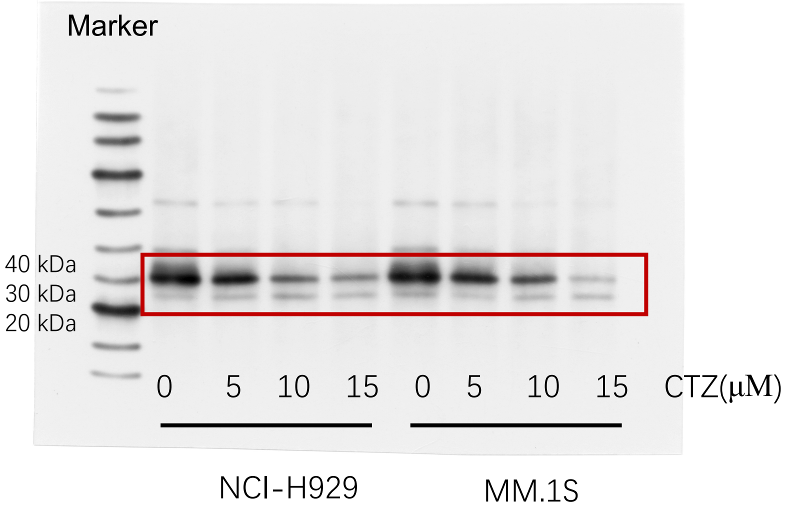

p-IκBα(3) 40kDa

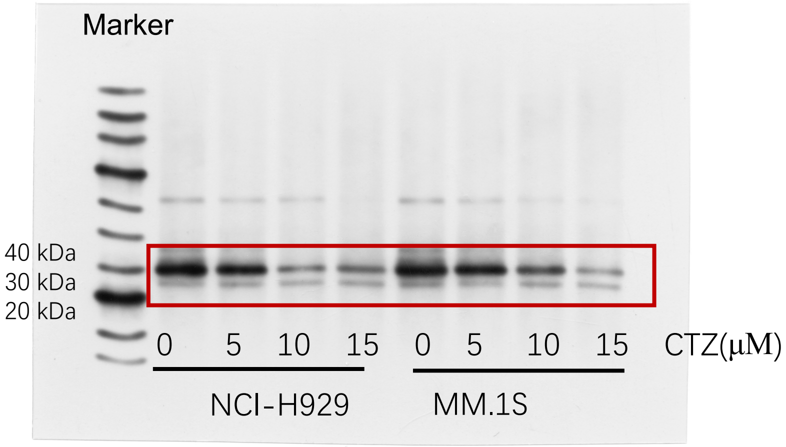

p65(1) 65kDa

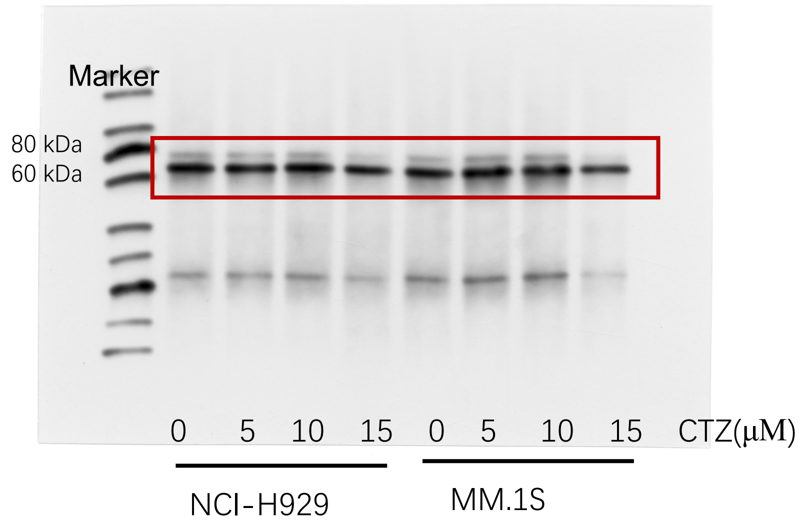

p65(2) 65kDa

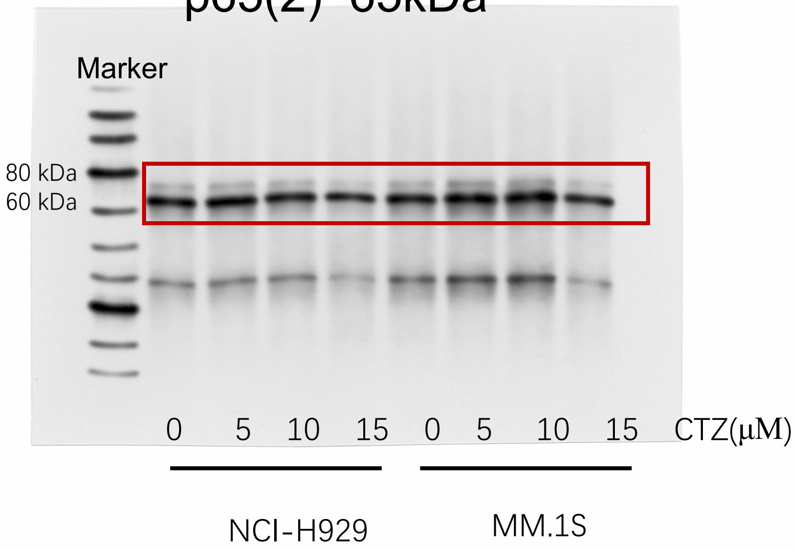

p65(3) 65kDa

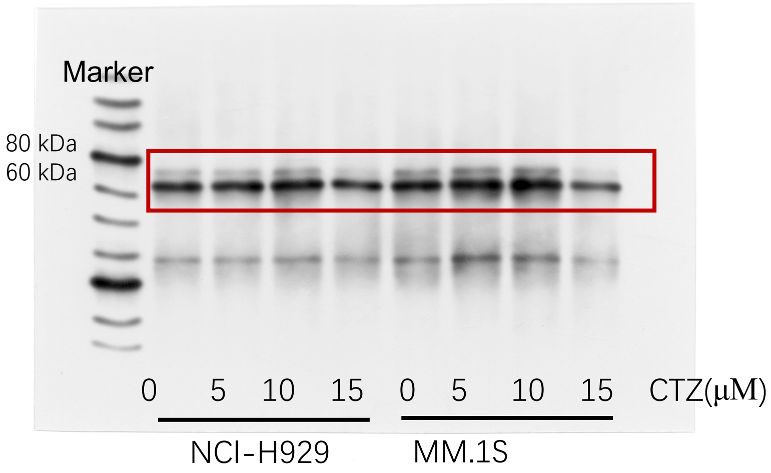

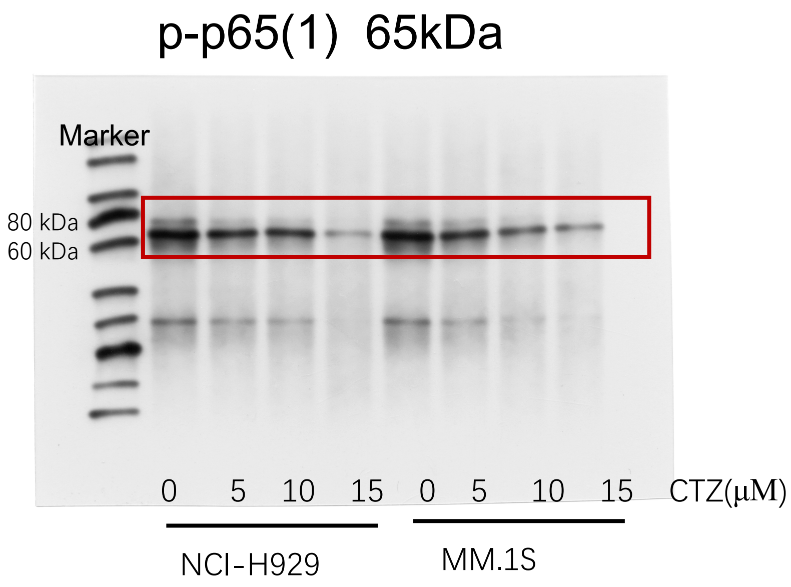
p-p65(1) 65kDa
Marker
80 kDa
60 kDa
0 5 10 15 0 5 10 15 CTZ(μM)
NCI-H929
MM.1S

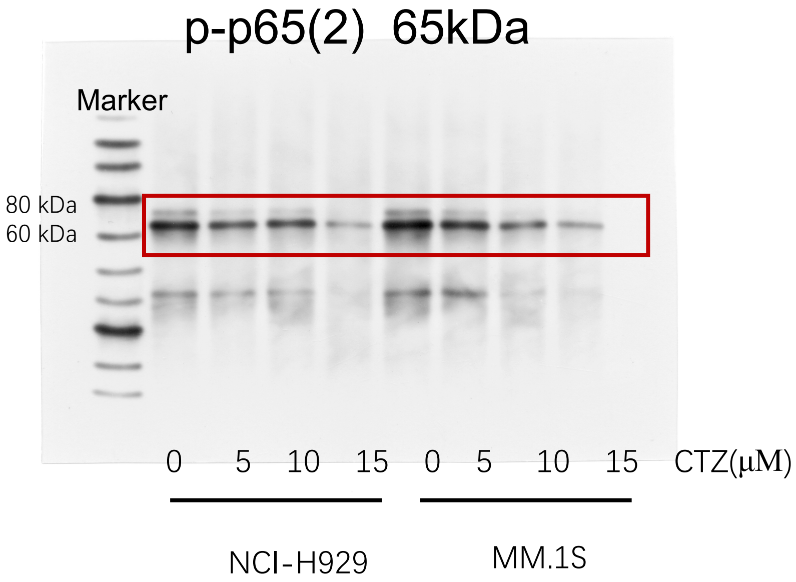
p-p65(2) 65kDa
Marker
80 kDa
60 kDa
0 5 10 15 0 5 10 15 CTZ(μM)
NCI-H929
MM.1S

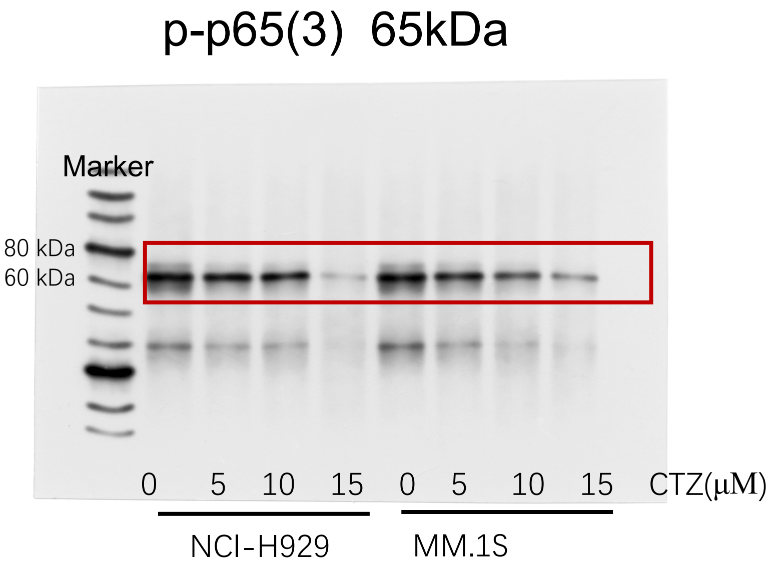
p-p65(3) 65kDa
Marker
80 kDa
60 kDa
0 5 10 15 0 5 10 15 CTZ(μM)
NCI-H929
MM.1S

β-actin(1) 45kDa

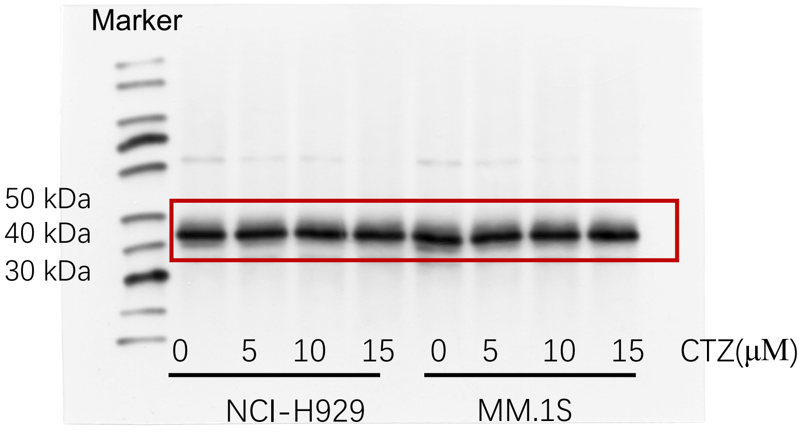

β-actin(2) 45kDa

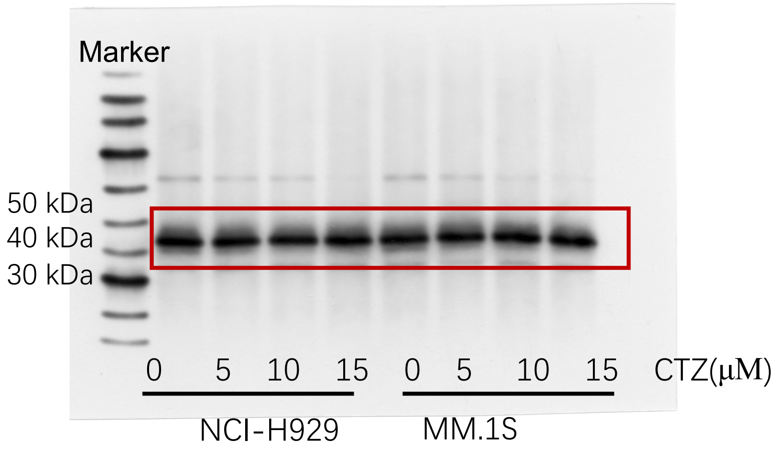

β-actin(3) 45kDa

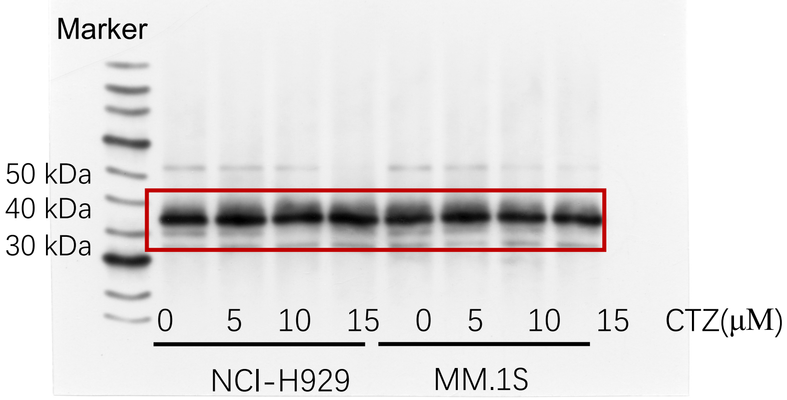

Supplement: Supplementary file 3 — Supplementary Information 3. [file 41598_2024_66367_MOESM3_ESM.pdf]
