# Supplementary material for: Clotrimazole inhibits growth of multiple myeloma cells in vitro via G0/G1 arrest and mitochondrial apoptosis
Source: Sci Rep. 2024 Jul 4;14:15406. doi: 10.1038/s41598-024-66367-5 (PMC11224322; doi:10.1038/s41598-024-66367-5)
Supplement: Supplementary file 4 — Supplementary Figure 1. [file 41598_2024_66367_MOESM4_ESM.docx]

Supplementary Figure 1. Effect of Clotrimazole (CTZ) treatment on cell viability of different multiple myeloma cell lines. This figure demonstrates the effect of CTZ treatment on cell viability of four multiple myeloma cell lines (a. MM.1S, b. NCI-H929, c. KMS-11, d. U266) after 24 and 48 hours. Cell viability was assessed by cell proliferation assays (CCK-8), and the results were expressed as percentage of cell viability. In each figure, solid dots and squares represent the results of 24 and 48 hour treatments, respectively. Each data point represents the mean ± standard Deviation (SD) of three independent experiments.
